# Systemic Lupus Erythematosus in a Nonagenarian Woman: A Case Report

**DOI:** 10.7759/cureus.69812

**Published:** 2024-09-20

**Authors:** Noelia Rodriguez-Perez

**Affiliations:** 1 Internal Medicine-Rheumatology, University of Puerto Rico, Medical Sciences Campus, San Juan, PRI

**Keywords:** late-onset lupus, late-onset sle, nonagenarian, pleuritis, sle, systemic lupus erythematosus

## Abstract

In this case report, we present an elderly Hispanic woman with systemic lupus erythematosus (SLE) exhibiting a unique presentation characterized by a substantial unilateral pleural effusion, cardiac effusion, nephrotic-range proteinuria, and arthritis. The patient demonstrated improvement after receiving intravenous corticosteroids, followed by oral corticosteroids and a low dose of azathioprine. This case underscores the atypical presentation of SLE in the elderly, involving major organ systems, and highlights the patient's stability after three years of low immunosuppressive treatment.

## Introduction

Systemic lupus erythematosus (SLE) is a chronic autoimmune disease that can affect multiple organs and has a diverse clinical presentation. Late-onset SLE represents a subgroup of SLE that can be seen in 10.63% of patients [1]. Disease diagnosis in this group may be challenging and delayed. The clinical presentation has been described as milder, although the course of their disease may be more aggressive than in younger patients [2-4]. They present with fewer renal manifestations, cytopenia, and mucocutaneous involvement [1,2]. Serositis may be seen more often in older age [5]. Another study done in older patients showed other findings, being in this group of patients older than 80 years, joint skin and hematological manifestations were the most common [6]. Positive Smith antibody, ribonucleoprotein, and hypocomplementemia are less common in the late-onset group. Ro and La antibody positivity is more frequent in older patients, whereas anti-SM and anti-DNA antibodies are more commonly seen in younger patients. The late-onset group received less aggressive treatment but had higher mortality most likely due to cardiovascular risk and the aging process [1,4].

## Case presentation

This 90-year-old woman, with a medical history notable for arterial hypertension, hypothyroidism, generalized osteoarthritis, and peripheral venous insufficiency, presented with recurrent unilateral pleural effusion and nephrotic-range proteinuria. Her daily medications included levothyroxine 88 mcg and enalapril 2.5 mg. She maintained independence in her activities until two months before admission, reporting worsening arthralgias, leg swelling, and an intermittent productive cough. Initially attributing these symptoms to pre-existing conditions, she sought evaluation due to new-onset exertional shortness of breath.

**Figure 1:**
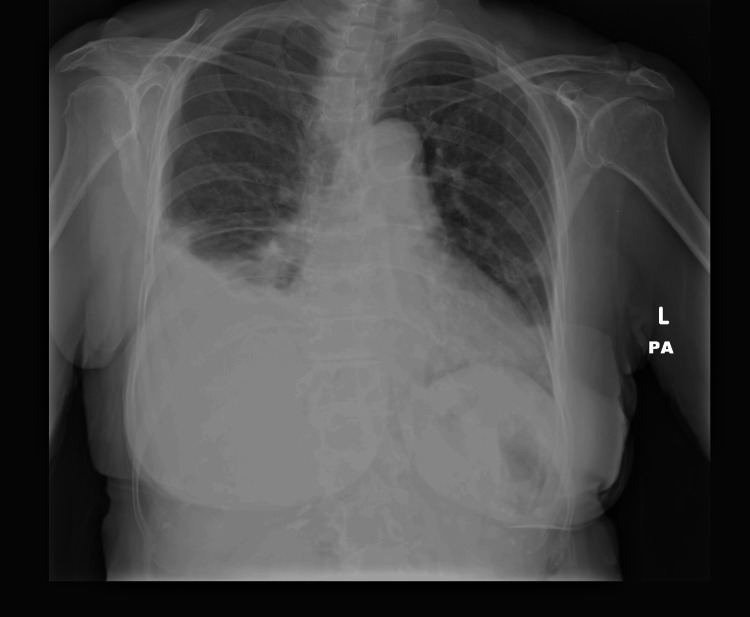
Chest X-ray. Large right pleural effusion.

Upon arrival at the emergency room, vital signs were as follows: temperature at 37°C, heart rate at 76 beats per minute, blood pressure at 130/90 mmHg, respiratory rate at 17 breaths per minute, and pulse oximeter saturation at 92%. Physical examination revealed decreased right lung sounds and mild pitting edema in both legs. Initial laboratory findings included normocytic, normochromic anemia (hemoglobin = 11 grams per deciliter), and significant proteinuria (>1 gram for a 24-hour collection). White blood cell count, platelet levels, fasting glucose, thyroid, and renal and liver function tests were within normal limits. Imaging revealed a large right pleural effusion with compressive atelectasis of the right lobe on chest X-ray (Figure 1) and chest CT.

Thoracentesis yielded bloody pleural fluid (exudative effusion, Light’s criteria ratio = 0.7) with negative cultures and cytology. COVID, influenza, and mycoplasma tests were negative. Empirical broad-spectrum antibiotics were initiated to cover for community-acquired pneumonia.

Cardiac and malignancy considerations were explored. Cardiac enzymes were normal, EKG showed no acute changes, and echocardiogram revealed a right pleural effusion and small pericardial effusion. CT scans showed no lymphadenopathy or masses. A recent mammogram displayed only benign changes. Despite initial improvement, symptoms recurred, necessitating three thoracentesis within the first two weeks. Proteinuria persisted at approximately 2 g in 24-hour urine collection, but the patient declined a renal biopsy. Further serological evaluation revealed a positive anti-nuclear antibody (ANA) at a titer of 1:320 by indirect immunofluorescence with a speckled pattern and perinuclear anti-neutrophil cytoplasmic antibody (p-ANCA). Other autoantibodies were negative, and complement levels (C3, C4) were within normal limits. SLE was diagnosed based on positive ANA, pleural and pericardial effusions, and marked proteinuria.

The initial treatment was with intravenous corticosteroids (methylprednisolone) 1 mg/kg and azathioprine 50 mg twice daily. Hydroxychloroquine was contraindicated due to prior retinal problems. After one week, pleural effusion decreased, and proteinuria improved from 1 g to 350 mg in 24 hours. The patient transitioned to oral corticosteroids (prednisone) tapered to 5 mg daily, and azathioprine was discontinued after six months due to gastrointestinal side effects. Two years later, the patient remains in complete remission using only low-dose prednisone (5 mg daily).

## Discussion

This case highlights the importance of considering the development of an autoimmune disease like SLE in very elderly patients. Late-onset lupus is used to describe the condition diagnosed in patients ≥ 50 years of age in the medical literature. The group of patients who fall into this category tends to differ in both clinical manifestations and disease progression when compared to early-onset one [1-4]. One of the characteristics that stands out is that the late-onset group can present more frequently with serositis but experience less renal and mucocutaneous involvement [1,2,7]. The available data are much smaller in patients who develop SLE at a very advanced age like our patient who began with symptoms at age 90. A cohort of patients who developed SLE after the age of 80 demonstrated that the most common manifestations in this group were joint, skin, and hematologic involvement but it was only a reduced number of patients [6]. The fact that our patient presented with pericardial and recurrent pleural effusions does not appear to be unusual at this age, but the fact that she had such marked proteinuria makes it a rare presentation.

The variations in the presentation of SLE across different age groups can be attributed to various factors. It is widely acknowledged that SLE manifestations are influenced by hormonal fluctuations in women over time [8]. In the post-menopausal stage, SLE typically presents milder symptoms. Additionally, age-related changes in the immune response to environmental factors may contribute to these differences. Previous studies have demonstrated that cells of the innate immune system also exhibit alterations in elderly individuals, indicating that both innate and adaptive immune system dysfunctions are critical in explaining the immune changes seen in aging. These alterations may contribute to an atypical progression of diseases or the development of other inflammatory conditions. Furthermore, significant reductions in the proliferation of regulatory T cells and the production of B11 cell growth factors have been observed, along with decreases in CD4 and CD8-positive T cell subpopulations and reduced expression of the CD28 coreceptor. This deficiency has been implicated in the diminished response to antigens and mitogens [9-11].

The patient described in this case had not recently initiated any new medications, experienced no recent infectious episodes, and had not received any vaccines that could serve as potential triggers for autoimmune disease development. It is probable that her symptoms had been manifesting for several months prior to admission, marked by increased fatigue and joint pain. In individuals of this age group, symptoms often overlap with comorbid conditions, making diagnosis challenging. For patients who do not show improvement with conventional treatments for their comorbid conditions, it is advisable to consider serologic studies. These tests can aid in identifying underlying autoimmune diseases that may not be readily apparent based solely on clinical symptoms.

Our patient only had positive ANA and p-ANCA tests. This serology is quite common in lupus patients. It has been reported that a high percentage of lupus patients may be antineutrophil cytoplasmic antibody (ANCA)-positive [12,13]. There are publications that support the absence of any relationship between having a positive ANCA and any specific lupus manifestation. However, there are others where a higher incidence of some clinical manifestations like renal involvement is associated with this antibody, predominantly p-ANCA [14]. The significance of the presence of the p-ANCA in elderly lupus patients is not clear. It is highly unlikely that our patient's manifestations were due to systemic vasculitis, as she met the classification criteria for SLE and did not present other features suggestive of ANCA-associated vasculitis.

Treatment recommendations for lupus nephritis are the same for early-onset lupus and late-onset lupus [15]. Usual treatment includes high-dose corticosteroids in addition to immunosuppressive medications. Patients in the late-onset SLE group may receive lower doses of glucocorticoids and immunosuppressive agents to reduce the frequency of adverse effects, given the higher prevalence of comorbid conditions. This should not alter the promptness and diligence with which these patients receive treatment because despite having a benign course of disease, they have poorer prognosis due to aging and vascular risk factors [1,2,4]. The patient of the case was treated with methylprednisolone 1 mg/kg in addition to a low azathioprine dose. She made rapid improvement and has been in remission for two years. This shows that rapid diagnosis and medical intervention can improve the prognosis of these patients who develop lupus in old age.

## Conclusions

In conclusion, this case report highlights the diagnostic and therapeutic challenges associated with late-onset SLE in a nonagenarian woman. The patient presented with a rare combination of major organ involvement, including substantial unilateral pleural effusion, cardiac effusion, nephrotic-range proteinuria, and arthritis. Her unique clinical presentation underscores the heterogeneity of SLE manifestations in the elderly population.

The patient's positive response to treatment, consisting of intravenous corticosteroids, oral corticosteroids, and a low dose of azathioprine, in this particular case, supports the effectiveness of prompt and tailored immunosuppressive therapy in achieving disease remission even in the very elderly. Importantly, the management of late-onset SLE requires a delicate balance, considering the higher prevalence of comorbidities, potential drug interactions, and increased susceptibility to infectious processes in this age group. Continued research and reporting of such cases are crucial for expanding our understanding of the disease spectrum and refining therapeutic strategies, especially in the context of an aging population.
